# Development of an Ultra-High Performance Liquid Chromatography Method for Simultaneous Determination of Six Active Compounds in *Fructus aurantii* and Rat Plasma and Its Application to a Comparative Pharmacokinetic Study in Rats Administered with Different Doses

**DOI:** 10.1155/2018/7579136

**Published:** 2018-05-10

**Authors:** Wenbo Wang, Linlin Zhao, Huiyong Huang, Jiamei Yao, Lu Zhou, Dongsheng Wang, Xinjian Qiu

**Affiliations:** ^1^Institute of Integrated Traditional Chinese and Western Medicine, Xiangya Hospital, Central South University, Changsha, Hunan, China; ^2^Physical Examination Center, The Third Xiangya Hospital, Central South University, Changsha, Hunan, China; ^3^Provincial Key Laboratory of TCM Diagnostics, Hunan University of Traditional Chinese Medicine, Changsha, Hunan, China; ^4^Department of Gerontology, Xiangya Hospital, Central South University, Changsha, Hunan, China

## Abstract

A rapid, accurate, and sensitive ultra-high performance liquid chromatography (UHPLC) method was established for simultaneously detecting naringin, hesperidin, neohesperidin, meranzin hydrate, naringenin, and hesperetin in *Fructus aurantii* (FA) decoction. Analysis was performed on Waters BEH (R) C18 (50 mm × 2.1 mm, 1.7 *μ*m) at a flow rate of 0.2 mL/min by using (A) acetonitrile and (B) 0.5% acetic acid-water as the mobile phase. The method was well validated on linearity, precision, recoveries, and stability. Then, we used the same UHPLC conditions for quantitative analysis of meranzin hydrate, naringenin, and hesperetin in rat plasma. The method proved to be linear within the concentration ranges of 3.3–3300 ng/mL for meranzin hydrate, 6.95–3555 ng/mL for naringenin, and 1.8–236 ng/mL for hesperetin. The RSD of precision ranged from 1.22% to 9.08%, and the average extraction recovery ranged from 96.49 ± 1.42% to 102.01 ± 3.16%. Besides, we performed a comparative pharmacokinetic study after oral administration of FA decoction at a low dose of 15 g/kg and high dose of 30 g/kg body weight for seven days to rats. The AUC_(0–*t*)_ and *C*_max_ of meranzin hydrate, naringenin, and hesperetin were multiplied significantly with the increase of FA dosage, and the *t*_1/2_ of meranzin hydrate was faster than naringenin and hesperetin in the two groups.

## 1. Introduction

*Fructus aurantii* (FA), also called *Zhi Qiao* in Chinese, refers to the dried unripe fruit of *Citrus aurantium* L. or its cultivar (Rutaceae). FA is a supremely important Qi-invigorating herb in China for centuries, and it vigorously modulates the motion of Qi and fortifies the spleen and stomach. Thus, FA is usually taken to remedy indigestion problems, like postprandial fullness, early satiation, abdominal pain, and distension, and other symptoms caused by the obstruction of the Qi activity [1]. In view of its definite effect, FA has been extensively applied to compose numerous complex Chinese prescriptions, like Chaihu-Shugan-San [2], and FA-Magnolia bark decoction [3]. It is well verified that the primary chemical compositions in FA are flavonoids, alkaloids, coumarins, and volatile oils [4, 5]. Flavonoid glycosides such as naringin, hesperidin, neohesperidin, naringenin, and hesperetin and their respective aglycones, polymethoxylated flavones, and coumarins, like meranzin hydrate, are all components found in FA [6–9]. Various work published in the literature have established that these compounds possess astoundingly biomedical properties, such as prokinetic effect [2], antidepression activity [10, 11], inflammation alleviating function [12], antioxidant and free-radical scavenging effect [13], anticarcinogenic activity [14, 15], antihypersensitivity [16], and hypoglycemic and hypolipidemic effects [17].

To date, all the published methods were developed for determining components in FA. Zhao et al. [18] used a HPLC-PDA method to identify 8 components (rutin, narirutin, naringin, hesperidin, neohesperidin, poncirin, nobiletin, and tangeretin) in FA, but this method was unreasonably time-consuming (60 min). Li et al. [19] developed a LC-MS/MS method to determine naringin, hesperidin, and neohesperidin in FA-medicated rat serum, whereas the author did not conduct pharmacokinetic experiments, and the chromatographic peak shape seemed imperfect. Zhang et al. [20] developed a HPLC method to determine naringin and neohesperidin in rat plasma; however, there was no research work on content determination. Cao et al. [21] simultaneously quantified six flavone glycosides (narirutin, naringin, hesperidin, neohesperidin, nobiletin, and tangeretin) in FA by UPLC-PDA, but they also did not carry out pharmacokinetic study. As mentioned above, there have been no reports focusing on quantitative determination of FA decoction and pharmacokinetic studies at the same time. In addition, our previous investigation suggested that both meranzin hydrate and FA had gastroprokinetic and antidepressant effect, and meranzin hydrate induced similar effect to FA on rat gut motility via activation of H1 histamine receptors [22]; moreover, meranzin hydrate could exert the antidepressant property by activating AMPA receptors, the ERK_1/2_ pathway, and subsequent modulation of brain-derived neurotrophic factor (BDNF) levels [23]. In functional dyspepsia patients, we determined meranzin hydrate in the plasma and conducted the pharmacokinetic study of meranzin hydrate after oral administration of Chaihu-Shugan-San [2]. Nevertheless, there are few papers published on the determination of meranzin hydrate not only in FA extract but also in rat plasma.

**Figure 1:**
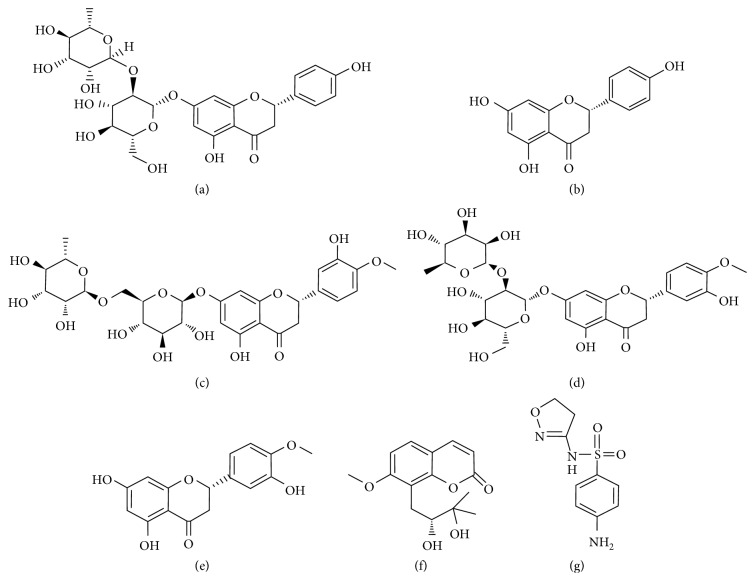
Chemical structures of (a) naringin, (b) naringenin, (c) hesperidin, (d) neohesperidin, (e) hesperetin, (f) meranzin hydrate, and (g) internal standard (IS, sulfamethoxazole).

Herein, a simple and accurate UHPLC detection method for simultaneous determination of six active compounds (naringin, naringenin, hesperidin, neohesperidin, hesperetin, and meranzin hydrate; Figure 1) in FA decoction was successfully established and well validated for quality evaluation. Then, we further used the same UHPLC chromatographic condition for quantitative analysis of meranzin hydrate, naringenin, and hesperetin in rat plasma and performed a comparative pharmacokinetic study after oral administration of FA decoction at a low dose of 15 g/kg and high dose of 30 g/kg body weight for seven days to rats.

## 2. Materials and Methods

### 2.1. Chemicals and Reagents

Standards including naringin, hesperidin, neohesperidin, naringenin, and hesperetin (purity >98%) were purchased from Chengdu Pufei De Biotech Co., Ltd. (Chengdu, China). Meranzin hydrate (purity >98%) was purchased from DIAO Company (Chengdu, China). Sulfamethoxazole (SMZ, purity >98%) was taken as an internal standard (IS) and from the National Institute for the Control of Pharmaceutical and Biological Products (Beijing, China). Acetonitrile and methanol (HPLC grade) were obtained from Sigma-Aldrich (St. Louis, MO, USA). Glacial acetic acid and ethyl alcohol (analytical grade) were obtained from Heng Xing Chemical Factory (Tianjin, China), and purified water was from Hangzhou Wahaha Group Co., Ltd. (Hangzhou, China). *β*-Glucuronidase was from EMD Millipore Co., Ltd. (Boston, Massachusetts, USA), and acetic acid buffer solution was from Dingguo Biotechnology Co., Ltd. (Beijing, China). FA (crude drug) was collected from Hunan province and bought and authenticated by Professor Sui-Yu Hu (Xiangya Hospital, Central South University, Hunan, China).

### 2.2. Instruments and Chromatographic Conditions

Analyses were implemented on a Waters ACQUITY UHPLC system and an ACQUITY PDA (Waters, Milford, MA, USA). The analytical column was a Waters BEH (R) C18 (50 mm × 2.1 mm, 1.7 *μ*m) from Waters Co. (USA) with temperature at 40°C. (A) Acetonitrile and (B) 0.5% acetic acid-water constituted the two parts of the mobile phase at the following gradient elution procedures (0–2 min, 20–20% A; 2–4 min, 20–30% A; 4–5 min, 30–30% A; 5–7 min, 30–40% A; 7–8 min, 40–40% A; and 8–10 min, 40–20% A). The flow rate was controlled at 0.2 mL/min, and the injection volume was 5 *µ*L.

### 2.3. Preparation of FA Decoction, Standard, and Quality Control (QC) Solutions

The raw herb (200 g) was immersed in distilled water (1 : 8, *w*/*v*) for 0.5 h at room temperature and thereafter was boiled for 0.5 h. The filtrate was collected, and the residue was boiled again in distilled water (1 : 6, *w*/*v*) for 0.5 h. The filtrates were mixed together, filtered through a 0.45 *µ*m porous filtration membrane, then farther concentrated in the rotary evaporator to a final concentration equivalent to 1.7 g/mL FA, and stored at −20°C until analyzed. In the experiment, FA decoction (100 *µ*L) was precisely measured, 900 *µ*L methanol was added, then ultrasound was done for 5 min, and it was centrifuged at 15000 rpm (4°C) for 10 min; the supernatant liquor (20 *µ*L) was shifted to a 1.5 mL EP tube, methanol-water (780 *µ*L) was added, and ultrasound was performed, and it was centrifuged at 15000 rpm (4°C) for 10 min again. Finally, 5 *µ*L of the supernatant liquor was injected into the apparatus for detection.

The concentrations of standard solutions were 1492 *µ*g/mL for naringin, 747 *µ*g/mL for hesperidin, 1496 *µ*g/mL for neohesperidin, 198 *µ*g/mL for meranzin hydrate, 128 *µ*g/mL for naringenin, and 273 *µ*g/mL for hesperetin. The concentrations of each analyte in standard mixture solutions were as follows: 497.30 *µ*g/mL for naringin, 124.50 *µ*g/mL for hesperidin, 374.00 *µ*g/mL for neohesperidin, 6.61 *µ*g/mL for meranzin hydrate, 2.13 *µ*g/mL for naringenin, and 5.69 *µ*g/mL for hesperetin. Then, the standard mixture solution was diluted to a suite of concentration (1/2, 1/4, 1/8, 1/16, 1/32, and 1/64) to establish calibration curves. QC samples were prepared at three concentration levels containing naringin (31.08, 124.33, and 497.30 *µ*g/mL), hesperidin (7.78, 31.13, and 124.50 *µ*g/mL), neohesperidin (23.38, 93.50, and 374.00 *µ*g/mL), meranzin hydrate (0.41, 1.65, and 6.61 *µ*g/mL), naringenin (0.13, 0.53, and 2.13 *µ*g/mL), and hesperetin (0.35, 1.40, and 5.60 *µ*g/mL).

### 2.4. Method Validation for FA Content Determination

We precisely injected 5 *µ*L sample from the above 7 concentrations of mixed liquor in order (3 replicates per concentration). Method validation was followed with Guidance for Industry Bioanalytical Method Validation by U.S. Food and Drug Administration (FDA). Limit of detection (LOD) under the analytical approach was measured at a signal-to-noise (S/N) ratio of 3. Lower Limit of Quantification (LLOQ) was defined as the lowest concentration of the standard curve that can be measured with a precision within 20% and an accuracy of 80–120%.

Intra- and interday variations were for determining the precision of the developed method. Relative standard deviation (RSD) was utilized as a measurement of precision. Intra- and interday repeatability were determined on 5 replicates within 1 day and 5 consecutive days, respectively.

The accuracy of the analytical method was evaluated by using the recovery test. Recoveries of 6 compounds were investigated by spiking with the authentic standards to the samples of FA before extraction. Five replicates of FA decoction were tested. Peak areas of each analyte in five FA samples were recorded. Then, the concentrations of the 6 compounds in FA decoction after spiking were calculated according to the peak area using the calibration curve. Average recovery percentage was calculated by the formula: recovery (%) = (total amount after spiking − original amount in sample)/spiked amount × 100%.

The stability of the analytical solution at environmental temperature was studied by detecting sample solution at 0, 2, 4, 8, 12, and 24 h. The RSD values of peak areas were taken for assessment.

### 2.5. Application in Pharmacokinetic Study

#### 2.5.1. Animals

16 male Sprague-Dawley (SD) rats weighing 200 ± 20 g were from experimental animal center of Central South University and fostered in an environmentally controlled room (temperature: 22–26°C, humidity: 40–60%, and 12 h dark-light cycle) for 1 week before the experiment. All experimental procedures were implemented strictly according to the guidelines of the Animal Care and Use Committee, Central South University, China. This research was approved by the Animal Ethics Committee of Central South University. Rats were randomly divided into 2 groups (*n*=8): low-dose group (15 g/kg FA decoction) and high-dose group (30 g/kg FA decoction). The two groups were given by gavage twice a day for 7 days in succession. At the 7th day of medication, the rats were prohibited feeding for about 12 hours without restriction of water. On day 8, the rats were orally administered for the last time at 8 am, and then plasma samples (0.5 mL) were collected from the caudal vein at 5, 10, 20, 30, 60, 90, 240, 360, 480, and 600 min into heparinized vacuum tubes, followed by centrifugation at 3500 rpm (4°C) for 10 min. Then, the plasmas were obtained and immediately stored at −20°C until analysis. During the experiment, the rats were injected intraperitoneally with physiological saline for maintaining fluid balance.

#### 2.5.2. Preparation of QC and Plasma Sample

Standard stock solutions were prepared by dissolving reference standards in methanol to yield concentrations of 198 *µ*g/mL for meranzin hydrate, 128 *µ*g/mL for naringenin, 273 *µ*g/mL for hesperetin, and 4.56 *µ*g/mL for SMZ. All solutions were stored at 4°C. QC samples were prepared at three concentration levels containing meranzin hydrate (6.5, 206, 3300 ng/mL), naringenin (13.9, 222, 3555 ng/mL), and hesperetin (3.7, 29.5, 236 ng/mL). Plasmas (100 *µ*L) were spiked with *β*-glucuronidase (5 *µ*L) and acetic acid buffer solution (10 *µ*L), subsequently vortexed for 10 seconds, and incubated for 4 h at 37°C. Then, SMZ (50 *µ*L) and methanol (135 *µ*L) were added, ultrasound was done for 10 min, and centrifugation at 12000 rpm (4°C) for 10 min was performed. The supernatant (50 *µ*L) was transferred, distilled water (50 *µ*L) was added, then ultrasound and centrifugation again. At last, the supernatant of 5 *µ*L was injected into UPLC.

#### 2.5.3. Method Validation for Pharmacokinetic Study

*(1) Specificity*. Specificity refers to analyze different plasma samples for interference at the retention time of the constituent. It was evaluated by comparing the chromatogram of blank plasma, blank plasma with the reference of three compounds, and medicated plasma.

*(2) Linearity*. Blank plasmas of healthy rats (7 copies, each 100 *μ*L) were added with suitable amount of stock solution of meranzin hydrate, naringenin, and hesperetin. Then, 50 *μ*L SMZ was added to each solution and a specific amount of methanol to a constant volume of 300 *μ*L, yielding a series of concentrations of 3300, 825, 206, 51.6, 12.9, 6.5, and 3.3 ng/mL for meranzin hydrate, 3555, 889, 222, 55.5, 27.8, 13.9, and 6.95 ng/mL for naringenin, 236, 59, 29.5, 14.7, 7.3, 3.7, and 1.8 ng/mL for hesperetin.

*(3) Precision and Accuracy*. The intra- and interday precisions were determined by assaying 5 replicates of QC samples at 3 different concentrations on the same day or three consecutive days. Precision was estimated through intra/interday RSD, and accuracy was weighed as a relative error (RE).

*(4) Extraction Recovery*. The extraction recoveries of the three constituents were evaluated by comparing the relative peak areas obtained from plasma samples with the constituents spiked before and after extraction.

*(5) Stability*. QC plasma samples of each concentration at low, medium, and high levels were detected. The freeze and thaw stability were investigated after three freeze (−20°C) and thaw (room temperature) cycles on consecutive days. Long-term stability was studied by storing QC samples at −20°C for 20 days. Short-term stability was assessed by analyzing QC samples kept at room temperature for 4 h.

### 2.6. Statistical Analysis

The database was set up with the SPSS 17.0 software package from SPSS Inc., Chicago, Illinois (US). Differences between the two groups were analyzed by one-way analysis of variance. A two-tailed *P* value of <0.05 was considered statistically significant. The DAS 3.2.8 pharmacokinetic program (Chinese Pharmacological Society) was used to process pharmacokinetic parameters.

## 3. Results and Discussion

### 3.1. Content Determination in FA Decoction

#### 3.1.1. Analysis Method Optimization

In our study, various mobile phase conditions were tried to obtain optimal responses, suitable retention times, and good peak shapes. Methanol-water, acetonitrile-water, even adding different concentrations of phosphoric acid and acetic acid, 0.5% acetic acid-100% water were all tested as potential mobile phases. The different chromatographic system, gradient elution program, flow rate, and over temperature were also optimized. Eventually, a gradient elution by acetonitrile and 0.5% acetic acid-water with a flow rate of 0.2 mL/min at 40°C was chosen to obtain satisfactory sensitivity and good peak shapes.

**Figure 2:**
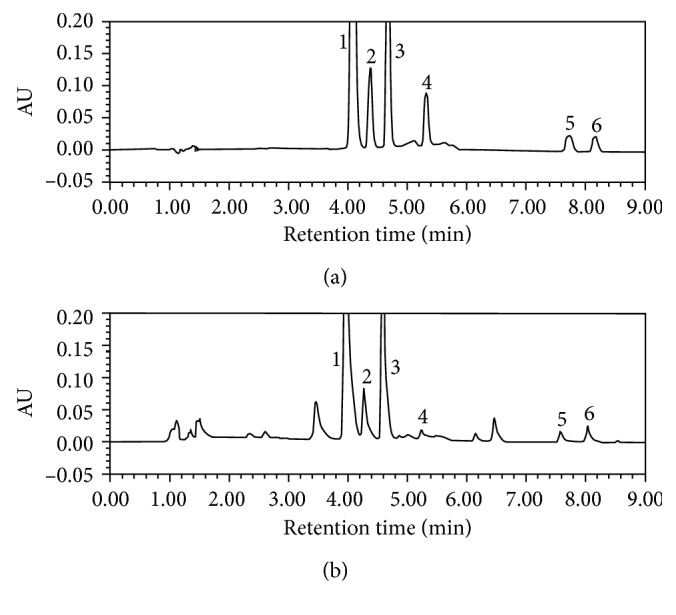
(a) Typical chromatogram of the standard mixture, containing 124.33 *μ*g/mL for naringin, 31.13 *μ*g/mL for hesperidin, 93.50 *μ*g/mL for neohesperidin, 1.653 *μ*g/mL for meranzin hydrate, 0.533 *μ*g/mL for naringenin, and 1.423 *μ*g/mL for hesperetin. (b) Typical chromatogram of FA decoction sample diluted for 400 times at 284 nm, containing 63.75 *μ*g/mL for naringin, 12.48 *μ*g/mL for hesperidin, 35.70 *μ*g/mL for neohesperidin, 0.875 *μ*g/mL for meranzin hydrate, 0.675 *μ*g/mL for naringenin, and 0.475 *μ*g/mL for hesperetin. 1: naringin; 2: hesperidin; 3: neohesperidin; 4: meranzin hydrate; 5: naringenin; 6: hesperetin.

To achieve the goal of strong absorption and low interference of components, peak area under the 284 nm wavelength was calculated for naringin, hesperidin, neohesperidin, naringenin, hesperetin, and 324 nm for meranzin hydrate. Under the proposed chromatographic conditions, all 6 constituents were sufficiently resolved and successfully separated within 9.0 min without “cross-talk” peaks. Representative chromatograms in blank solvent, standard solution, and FA decoction are shown in Figure 2. There was no endogenous peak interference present. The retention times of naringin, hesperidin, neohesperidin, meranzin hydrate, naringenin, and hesperetin were 4.12, 4.39, 4.71, 5.30, 7.62, and 8.15 min, respectively.

#### 3.1.2. Method Validation for FA Content Determination

*(1) Calibration Curves, LOD, and LLOQ*.  Table S1 (in Supplementary Material) presented the calibration equations, coefficients (*r*), linear range, LOD, and LLOQ of 6 components. All calibration curves exhibited good linear relationship, and *r* were higher than 0.9939.

*(2) Precision, Recovery, and Stability*. Results concerning the precision of this developed method were exhibited in Table S2. Overall intraday variation of 6 components was 2.56% to 7.14%, and interday precision was 2.38% to 6.67%, which suggested that the corresponding assay method had good precision. It was reliable and reproducible for content determination of the 6 components.

Table S3 showed the recovery tests. The average recoveries of all 6 tested compounds were within the range of 96.89–101.20%, with an RSD value varying from 2.27 to 5.09% (*n*=5), which demonstrated that the recoveries were consistent, accurate, and reproducible for the measurement.

Table S4 showed the stability tests. RSD values of the 6 compounds were below 2.64%. The data confirmed that there was no significant degradation, and all the 6 compounds were stable in the solution at least for 24 h.

#### 3.1.3. Content Determination of Six Constituents in FA Decoction

The newly developed analytical method was subsequently applied to determine the six constituents in FA decoction. The contents in FA decoction (per 1.7 g/mL) were naringin 24.84 ± 0.59 mg/mL, hesperidin 5.16 ± 0.18 mg/mL, neohesperidin 15.00 ± 0.76 mg/mL, meranzin hydrate 0.35 ± 0.01 mg/mL, naringenin 0.28 ± 0.01 mg/mL, and hesperetin 0.20 ± 0.01 mg/mL, and the overall RSD was below 5.10% (Table 1). There were significant differences in the content of the 6 components. Naringin is the most abundant constituent, followed by neohesperidin and hesperidin. The proposed method may be useful to the quality control of FA decoction.

### 3.2. Plasma Pharmacokinetic Study

#### 3.2.1. Plasma Sample Optimization

To select the best suitable IS, we tried different chemical compositions, such as caffeine, luteolin, diphenhydramine, and SMZ. Then, SMZ was selected as the IS of the three compounds in plasma sample determination for its stable, high recovery rate, and similarity in the retention. In order to establish an appropriate plasma processing method, different extraction methods of plasma samples—ethyl acetate, methanol, diethyl ether, acetonitrile and water bath, and adding acid or alkali—were all tested to examine the extraction effect of meranzin hydrate, naringenin, and hesperetin in plasma. When utilizing acetonitrile and water bath to eliminate protein of plasma samples, few chromatographic peaks existed, and even the target component did not appear. Similarly, by using methanol to precipitate protein, naringenin and hesperetin could not be detected. Besides, *β*-glucuronidase and acetate buffer were tried and added into plasma samples, vortexed, and incubated at 37°C for 30 seconds; then, SMZ solution and methanol were added. The method successfully elicited out the peaks of meranzin hydrate, naringenin, and hesperetin, and endogenous substances did not intervene the target peak. We also measured the extraction effect of incubation for 2 h, 4 h, 6 h, 12 h, and 24 h, respectively, and found that incubation for 4 h had the similar effect as 6 h, 12 h, and 24 h. Eventually, we chose *β*-glucuronidase and acetate buffer for enzymatic deconjugation, methanol for deproteinization, and incubation for 4 h as the optimal plasma pretreatment.

The optimized UHPLC method for content determination in FA decoction with the same mobile phase condition, the same chromatographic system and gradient elution program, and the same flow rate and temperature was also applied to the pharmacokinetic study of meranzin hydrate, naringenin, and hesperetin in rat plasma following oral administration of FA decoction at a dose of 15 g/kg and 30 g/kg body weight.

#### 3.2.2. Method Validation for Pharmacokinetic Study

**Figure 3:**
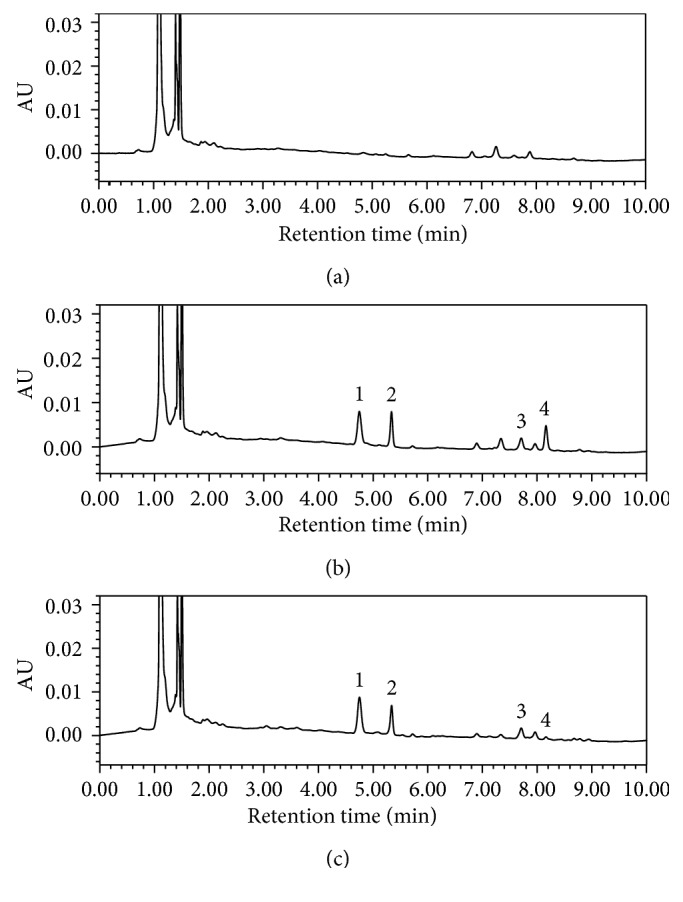
Typical chromatograms of the blank plasma (a), the blank plasma sample spiked with analytes (b), and plasma sample of 30 min following oral administration of FA decoction (c) at 284 nm. 1: SMZ; 2: meranzin hydrate; 3: naringenin; 4: hesperetin.

*(1) Specificity*. The typical chromatograms of blank plasma added with the 3 analytes and IS, and plasma sample after oral administration of FA decoction for 0.5 h (15 g/kg body weight) are represented in Figure 3. The retention times were about 4.62 min for SMZ, 5.34 min for meranzin hydrate, 7.79 min for naringenin, and 8.11 min for hesperetin. Due to the high selectivity of UHPLC, no significant endogenous components could interfere with the 3 constituents and SMZ.

*(2) Linearity of Calibration Curves and LLOQ*. As shown (Table 2), the method proved to be linear within the concentration ranges of 3.3–3300 ng/mL of meranzin hydrate, 6.95–3555 ng/mL of naringenin, and 1.8–236 ng/mL of hesperetin, respectively. The regression equations of the 3 constituents were as follows: *y* = 63.086*x* − 0.9615 (*r*=0.9989, meranzin hydrate), *y* = 210.36*x* + 8.3364 (*r*=0.9981, naringenin), and *y* = 29.540*x* + 3.5492 (*r*=0.9956, hesperetin). LLOQ for the 3 constituents in rat plasma were meranzin hydrate (3.30 ng/mL), naringenin (6.95 ng/mL), and hesperetin (1.80 ng/mL).

*(3) Precision and Accuracy*. In Table 3, the RSD of intraday precision ranged from 1.36% to 9.01%, and RE ranged from −7% to 2.2%; interday precision ranged from 1.22% to 9.08%, and RE ranged from −3.4% to 2.8%.

*(4) Recovery*. The average extraction recovery ranged from (96.49 ± 1.42)% to (102.01 ± 3.16)%, and the RSDs were less than 6.05% (Table 4), indicating that the method could ensure the acquisition of accurate and consistent data for all the constituents at different concentrations.

*(5) Stability*. Results of the stability tests in Table 4 showed that all constituents remained generally stable in plasma samples within three freeze-thaw cycles (RSD <3.57%), for 4 h at room temperature (RSD <3.42%), and for 20 days at −20°C (RSD <3.95%).

#### 3.2.3. Results of Pharmacokinetics

**Figure 4:**
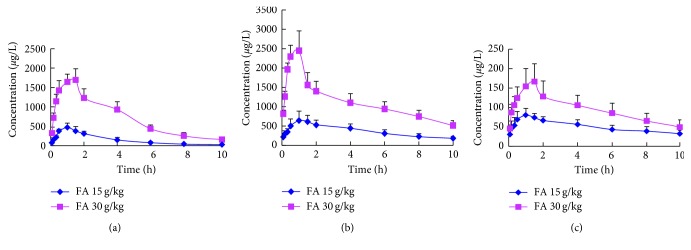
The mean (±SD, *n*=8) plasma concentration-time profiles for meranzin hydrate (a), naringenin (b), and hesperidin (c) following oral administration of FA decoction at doses of 15 g/kg and 30 g/kg.

The validated method was applied to determine the plasma concentrations of meranzin hydrate, naringenin, and hesperetin in rats after oral administration of FA decoction at a dose of 15 g/kg and 30 g/kg body weight. The mean plasma concentration-time profiles of meranzin hydrate, naringenin, and hesperetin are shown in Figure 4, and the estimated pharmacokinetic parameters are illustrated in Table 5. According to DAS software, the optimal metabolism process of the 3 components in rats could be depicted as a two-compartment pharmacokinetic model.

As shown, naringenin was the component with the highest concentration among all constituents in rat plasma, and this was identical with the Tong et al. study [9], for the reason that naringenin was a metabolite of naringin composed of the majority compound in FA decoction. The *C*_max_ of meranzin hydrate was inferior to that of naringenin. Also, hesperetin presented quite low concentrations in rat plasma, with the *C*_max_ at 83.90 ± 14.18 *μ*g/mL for the 15 g/kg group and 174.32 ± 39.04 *μ*g/mL for the 30 g/kg group, respectively.

The *t*_1/2*z*_ of meranzin hydrate was 2.67 ± 0.81 h (at the 15 g/kg group) and 2.23 ± 0.59 h (at the 30 g/kg group), and the elimination rate of meranzin hydrate was faster than naringenin (8.59 ± 9.25 h, 5.67 ± 2.90 h) and hesperetin (8.05 ± 3.10 h, 6.56 ± 3.35 h). Compared with the 15 g/kg group, oral administrationof 30 g/kg of FA, the AUC_(0–*t*)_ of meranzin hydrate, naringenin, and hesperetin was 4.8-fold, 3.05-fold, and 1.88-fold elevated, respectively; and *C*_max_ of meranzin hydrate, naringenin, and hesperetin was 3.62-fold, 3.72-fold, and 2.08-fold elevated, respectively. Besides, CL/F of meranzin hydrate, naringenin, and hesperetin was decreased, but *T*_max_ and MRT of the three components failed to have significant change. Results indicated that, as the dosage of FA increased, the plasma concentration of meranzin hydrate, naringenin, and hesperetin in plasma multiplied.

In this paper, we tested the quantity of 6 kinds of constituents (naringin, hesperidin, neohesperidin, meranzin hydrate, naringenin, and hesperetin) in FA decoction. However, naringin, hesperidin, and neohesperidin could not been detected in the rat plasma even after orally administering 30 g/kg FA decoction, which might be associated with the hydrolysis caused by microbial bacterial from the gastrointestinal tract [24, 25]. Naringin can be quickly absorbed into the body and also quickly eliminated after oral administration, and then metabolized into naringenin and naringenin glucuronide. Because of the similar structure with that of naringin, hesperidin was also quickly transferred to hesperetin [26].

Earlier research of our team has found that meranzin hydrate could accelerate gastrointestinal motility in rats subjected to the forced swimming test and reverse the inhibition effect of the decreased ghrelin levels on gastrointestinal motility, demonstrating that meranzin hydrate possessed antidepressive and prokinetic-like effects [23]. Besides, other report unveiled that naringenin and hesperetin contributed most for the significant galvanizing impact on small intestinal propulsion [27]. Therefore, by coordinating the pharmacodynamic studies with our pharmacokinetic observation, we may speculate that meranzin hydrate, naringenin, and hesperetin are the key components for improving gastrointestinal tract disorders. The pharmacokinetic study is not only beneficial for providing scientific basis for the safety and efficacy of clinical application but also a reference to the apprehension of the therapeutic mechanisms.

## 4. Conclusion

To the best of our knowledge, this is the first report using the same UHPLC chromatographic condition for determination of naringin, hesperidin, neohesperidin, meranzin hydrate, naringenin, and hesperetin in FA decoction and for detection of meranzin hydrate, naringenin, and hesperetin in rat plasma. Both of the detections were fully and well validated. Most importantly, we further performed the pharmacokinetics after oral administration of 15 g/kg and 30 g/kg FA decoction to rats. The AUC_(0–*t*)_ and *C*_max_ of meranzin hydrate, naringenin, and hesperetin multiplied significantly with the increase of FA dosage. The *t*_1/2_ of meranzin hydrate was faster than that of naringenin and hesperetin in the two groups. This work, on the one hand, demonstrated a detailed research of quantitative determination in FA decoction and rat plasma, as well as pharmacokinetics; on the other hand, it may lay the experimental foundation for pharmacokinetic interactions between FA and other drugs.

## Figures and Tables

**Table 1:** Contents of six components in FA decoction (1.7 g/mL; *n*=3).

Components	Contents (mg/mL)			Mean ± SD (mg/mL)	RSD (%)
	FA 1	FA 2	FA 3		
Naringin	25.50	24.69	24.35	24.84 ± 0.59	2.37
Hesperidin	4.99	5.13	5.35	5.16 ± 0.18	3.52
Neohesperidin	14.28	15.81	14.92	15.00 ± 0.76	5.10
Meranzin hydrate	0.35	0.34	0.36	0.35 ± 0.01	2.33
Naringenin	0.27	0.27	0.29	0.28 ± 0.01	4.87
Hesperetin	0.19	0.20	0.20	0.20 ± 0.01	2.83

**Table 2:** Regression equations, correlation coefficients, linear ranges, LOD, and LLOQ of meranzin hydrate, naringenin, and hesperetin in rat plasma.

Components	Linear regression equation	Correlation coefficient (*r*)	Linear range (ng/mL)	LOD (ng/mL)	LLOQ (ng/mL)
Meranzin hydrate	*y* = 63.086*x* − 0.9615	0.9989	3.3–3300	0.498	3.30
Naringenin	*y* = 210.36*x* + 8.3364	0.9981	6.95–3555	1.082	6.95
Hesperetin	*y* = 29.540*x* + 3.5492	0.9956	1.8–236	0.211	1.80

*y*: peak area of the components; *x*: concentration in ng/mL.

**Table 3:** Intraday and interday precisions and accuracies of meranzin hydrate, naringenin, and hesperetin in rat plasma (*n*=5).

Components	Spiked concentration (ng/mL)	Intraday			Interday		
		Observed concentration (ng/mL)	Precision (RSD, %)	Accuracy (Re, %)	Observed concentration (ng/mL)	Precision (RSD, %)	Accuracy (Re, %)
Meranzin hydrate	6.5	6.30 ± 0.43	6.82	−3	6.28 ± 0.57	9.08	−3.4
	206.0	203.15 ± 5.98	2.94	−1.3	211.84 ± 4.81	2.27	2.8
	3300.0	3290.75 ± 30.54	1.57	−2.5	3293.31 ± 32.25	1.39	−1.2

Naringenin	13.9	13.13 ± 1.64	5.44	−2.5	13.43 ± 1.23	3.66	2.2
	222.0	220.52 ± 6.88	6.71	2.2	219.88 ± 5.11	4.79	2.7
	3555.0	3550.48 ± 33.15	1.36	−1.9	3352.05 ± 35.08	1.22	−1

Hesperetin	3.7	3.44 ± 0.31	9.01	−7	3.64 ± 0.29	7.97	−1.6
	29.5	27.83 ± 2.54	5.50	−3.1	28.12 ± 2.25	5.12	2.4
	236.0	232.23 ± 7.68	3.36	−3	235.64 ± 7.25	2.22	−0.6

**Table 4:** Extraction recoveries and stability of meranzin hydrate, naringenin, and hesperetin in rat plasma.

Components	Spiked concentration (ng/mL)	Recovery (*n*=3)		Short-term stability		Freeze-thaw stability		Long-term stability	
		Mean ± SD (%)	RSD (%)	Mean ± SD (ng/mL)	RSD (%)	Mean ± SD (ng/mL)	RSD (%)	Mean ± SD (ng/mL)	RSD (%)
Meranzin hydrate	6.5	99.38 ± 1.18	2.01	6.31 ± 0.75	1.18	6.26 ± 0.53	0.85	6.38 ± 1.36	2.13
	206.0	101.81 ± 3.02	2.05	207.84 ± 46.76	2.25	210.11 ± 51.48	2.45	201.56 ± 71.96	3.57
	3300.0	102.01 ± 3.16	2.15	3305.34 ± 335.72	2.24	3310.91 ± 357.30	2.39	3297.28 ± 349.19	3.67

Naringenin	13.9	96.78 ± 1.20	6.05	13.03 ± 2.28	1.20	13.44 ± 4.79	2.74	13.32 ± 5.05	1.87
	222.0	98.25 ± 1.55	2.96	217.05 ± 22.66	2.06	219.51 ± 35.50	2.64	220.06 ± 34.22	2.53
	3555.0	98.96 ± 2.22	1.12	3549.93 ± 455.39	3.35	3552.68 ± 409.74	3.57	3550.10 ± 450.62	3.95

Hesperetin	3.7	96.49 ± 1.42	2.80	3.63 ± 0.87	2.40	3.52 ± 0.43	1.23	3.67 ± 0.57	1.55
	29.5	98.05 ± 2.45	3.03	27.88 ± 3.50	2.61	28.35 ± 4.03	2.71	28.28 ± 1.65	1.15
	236.0	99.17 ± 1.14	2.70	234.18 ± 22.13	3.42	235.03 ± 19.71	3.09	235.66 ± 14.53	2.01

**Table 5:** Pharmacokinetic parameters for meranzin hydrate, naringenin, and hesperetin in rat plasma after oral administration of FA decoction at doses of 15 g/kg and 30 g/kg (*n*=8).

Parameter	Meranzin hydrate		Naringenin		Hesperetin	
	15 g/kg group	30 g/kg group	15 g/kg group	30 g/kg group	15 g/kg group	30 g/kg group
AUC_(0–*t*)_ (*μ*g/L·h)	1534.84 ± 252.10	7370.77 ± 887.03^∗^	3662.98 ± 860.83	11176.77 ± 1413.17^∗^	508.31 ± 88.02	955.15 ± 226.98^∗^
MRT_(0–*t*)_ (h)	2.77 ± 0.34	3.21 ± 0.22	4.06 ± 0.33	3.89 ± 0.21	4.27 ± 0.27	4.15 ± 0.35
*t*_1/2*z*_(h)	2.67 ± 0.81	2.23 ± 0.59	8.59 ± 9.25	5.67 ± 2.90^∗^	8.05 ± 3.10	6.56 ± 3.35^∗^
*T*_max_ (h)	1.00 ± 0.27	1.25 ± 0.27	1.13 ± 0.35	0.81 ± 0.26	1.00 ± 0.38	1.31 ± 0.26
CL/F (L/h/kg)	9452.36 ± 1764.91	3875.84 ± 474.56^∗^	2816.64 ± 860.15	1986.11 ± 415.97^∗^	18224.53 ± 5230.06	21918.11 ± 6139.69^∗^
*C*_max_ (*µ*g/L)	504.75 ± 64.26	1827.68 ± 220.57^∗^	693.84 ± 208.24	2581.35 ± 388.30^∗^	83.90 ± 14.18	174.32 ± 39.04^∗^

^∗^ *P* < 0.05, compared to 15 g/kg FA group.

## Supplementary Materials

Supplementary Materials The detailed method validations for FA content determination are presented in the supplementary materials. The LC-MS chromatogram of FA decoction and its MS parameters are also included. Table S1: regression equations, correlation coefficients and linear ranges, and LOD and LLOQ of six components in FA decoction. Table S2: precision of six components in FA decoction (*n*=5). Table S3: spike recoveries of six components in FA decoction (*n*=5). Table S4: stability of six components in FA decoction (1.7 g/mL). Figure S1: LC-MS extracted total ion chromatogram (TIC) of 6 investigated compounds in the FA decoction sample (FA concentration at 250 *μ*g/mL) in the MRM scan mode. Table S5: the detected MS parameters for the six components.
