# Supplementary material for: Development of an Ultra-High Performance Liquid Chromatography Method for Simultaneous Determination of Six Active Compounds in Fructus aurantii and Rat Plasma and Its Application to a Comparative Pharmacokinetic Study in Rats Administered with Different Doses
Source: J Anal Methods Chem. 2018 May 10;2018:7579136. doi: 10.1155/2018/7579136 (PMC5971337; doi:10.1155/2018/7579136)
Supplement: Supplementary Materials — The detailed method validations for FA content determination are presented in the supplementary materials. The LC-MS chromatogram of FA decoction and its MS parameters are also included. Table S1: regression equations, correlation coefficients and linear ranges, and LOD and LLOQ of six components in FA decoction. Table S2: precision of six components in FA decoction (n=5). Table S3: spike recoveries of six components in FA decoction (n=5). Table S4: stability of six components in FA decoction (1.7 g/mL). Figure S1: LC-MS extracted total ion chromatogram (TIC) of 6 investigated compounds in the FA decoction sample (FA concentration at 250 μg/mL) in the MRM scan mode. Table S5: the detected MS parameters for the six components. [file 7579136.f1.doc]

**Journal of Analytical Methods in Chemistry**

**Supplementary material**

**Development of an Ultra High Performance Liquid Chromatography Method for Simultaneous Determination of Six Active Compounds in Fructus Aurantii, Rat Plasma and Its Application to a Comparative Pharmacokinetic Study in Different Doses Administrated Rats**

Wenbo Wang^1^, Linlin Zhao^2^, Huiyong Huang^3^, Jiamei Yao^4^, Lu Zhou^1^, Dongsheng Wang^1^, and Xinjian Qiu^1, 3^

^1^ Institute of Integrated Traditional Chinese and Western Medicine, Xiangya Hospital, Central South University, Changsha, Hunan, China.

^2^ Physical Examination Center, The Third Xiangya Hospital, Central South University, Changsha, Hunan, China.

^3^ Provincial Key Laboratory of TCM Diagnostics, Hunan University of Traditional Chinese Medicine, Changsha, Hunan, China.

^4^ Department of Gerontology, Xiangya Hospital, Central South University, Changsha, Hunan, China.

Correspondence should be addressed to Xinjian Qiu; qiuxjxy@csu.edu.cn.

Table S1 Regression equations, correlation coefficients and linear ranges, LOD and LLOQ of six components in FA decoction.

| Components | Linear regression equation | Correlation coefficient(r) | Linear range  (µg/mL) | LOD  (µg/mL) | LLOQ  (µg/mL) |
| --- | --- | --- | --- | --- | --- |
| naringin | *y* =2E-05*x*+1.8694 | 0.9999 | 7.78-479.30 | 1.252 | 7.78 |
| hesperidin | *y*= 3E-05*x*-0.6025 | 0.9974 | 1.95-124.50 | 0.299 | 1.95 |
| neohesperidin | *y*=2E-05*x*+0.8566 | 0.9997 | 5.84-374.00 | 0.922 | 5.84 |
| meranzin hydrate | *y*=1E-05*x*+0.1125 | 0.9939 | 0.10-6.61 | 0.013 | 0.10 |
| naringenin | *y*=1E-05*x*+0.0365 | 0.9972 | 0.03-2.13 | 0.003 | 0.03 |
| hesperetin | *y*=1E-05*x*+0.1418 | 0.9955 | 0.09-5.69 | 0.010 | 0.09 |

*y*: Peak area of the components; *x*: Concentration in µg/mL.

Table S2 Precision of six components in FA decoction (n=5)

| Components | Spiked conc.  (µg/mL) | Intra-day precision | | Inter-day precision | |
| --- | --- | --- | --- | --- | --- |
|  |  | Mean ± SD  (µg/mL) | RSD  (%) | Mean ± SD  (µg/mL) | RSD  (%) |
| naringin | 31.08 | 31.28 ±1.32 | 4.22 | 31.10 ±1.11 | 3.57 |
|  | 124.33 | 115.86 ± 2.97 | 2.56 | 118.36 ± 3.83 | 3.24 |
|  | 497.30 | 484.56 ±17.24 | 3.56 | 476.60 ± 22.07 | 4.63 |
| hesperidin | 7.78 | 7.46 ± 0.37 | 4.96 | 7.40± 0.34 | 4.59 |
|  | 31.13 | 32.79± 1.74 | 5.30 | 33.15 ±1.11 | 3.35 |
|  | 124.50 | 124.55 ±4.66 | 3.74 | 123.35± 4.98 | 4.04 |
| neohesperidin | 23.38 | 21.23±0.89 | 4.19 | 21.31 ± 0.91 | 4.27 |
|  | 93.50 | 90.48 ± 3.61 | 3.99 | 90.04 ± 3.69 | 4.10 |
|  | 374.00 | 357.21 ± 10.34 | 2.90 | 361.24 ± 14.26 | 3.95 |
| meranzin hydrate | 0.41 | 0.44 ± 0.02 | 4.55 | 0.42 ± 0.01 | 2.38 |
|  | 1.65 | 1.44 ± 0.04 | 2.78 | 1.48 ±0.07 | 4.73 |
|  | 6.61 | 6.27 ±0.17 | 2.71 | 6.25 ± 0.20 | 3.20 |
| naringenin | 0.13 | 0.14± 0.01 | 7.14 | 0.16±0.01 | 6.25 |
|  | 0.53 | 0.48 ± 0.02 | 4.17 | 0.49 ± 0.02 | 4.08 |
|  | 2.13 | 2.04 ± 0.10 | 4.90 | 2.20 ± 0.08 | 3.64 |
| hesperetin | 0.35 | 0.37 ± 0.02 | 5.40 | 0.36± 0.01 | 2.78 |
|  | 1.40 | 1.23 ± 0.05 | 4.06 | 0.60 ± 0.04 | 6.67 |
|  | 5.60 | 5.22 ± 0.19 | 3.64 | 5.24 ±0.21 | 4.01 |

Table S3 Spike recoveries of six components in FA decoction (n=5)

| Components | Sample  content(µg) | Added  amount(µg) | Measured  amount(µg) | Sample content(µg)  Mean ± SD | Measured amount(µg)  Mean ± SD | Recovery  (%) | RSD  (%) |
| --- | --- | --- | --- | --- | --- | --- | --- |
| naringin | 189.37 | 54.3 | 236.09 | 188.13 ± 1.13 | 240.75 ± 2.75 | 96.89 | 3.77 |
| hesperidin | 38.02 | 15.5 | 54.16 | 37.83 ± 0.45 | 53.52 ± 0.43 | 101.20 | 4.53 |
| neohesperidin | 110.56 | 25.7 | 135.12 | 110.81 ± 0.63 | 136.30 ± 1.00 | 99.16 | 2.27 |
| meranzin hydrate | 2.59 | 6.4 | 8.78 | 2.62 ± 0.068 | 8.87 ± 0.274 | 97.66 | 5.09 |
| naringenin | 1.98 | 2.4 | 4.31 | 1.99 ± 0.039 | 4.35 ± 0.105 | 98.40 | 4.54 |
| hesperetin | 1.45 | 3.5 | 4.92 | 1.48 ± 0.036 | 4.95 ± 0.096 | 99.39 | 2.31 |

Table S4 Stability of six components in FA decoction (1.7 g/mL)

| Components | Content(mg/mL) | | | | | | |
| --- | --- | --- | --- | --- | --- | --- | --- |
|  | 0h | 2h | 4h | 8h | 12h | 24h | RSD (%) |
| naringin | 25.24 | 25.35 | 24.92 | 25.45 | 24.98 | 25.15 | 0.82 |
| hesperidin | 4.95 | 4.83 | 4.71 | 5.02 | 4.87 | 4.82 | 2.21 |
| neohesperidin | 14.33 | 14.28 | 13.98 | 14.40 | 14.11 | 14.08 | 1.17 |
| meranzin hydrate | 0.34 | 0.36 | 0.33 | 0.34 | 0.35 | 0.34 | 2.64 |
| naringenin | 0.27 | 0.26 | 0.26 | 0.26 | 0.26 | 0.25 | 2.27 |
| hesperetin | 0.19 | 0.19 | 0.18 | 0.18 | 0.19 | 0.18 | 2.60 |


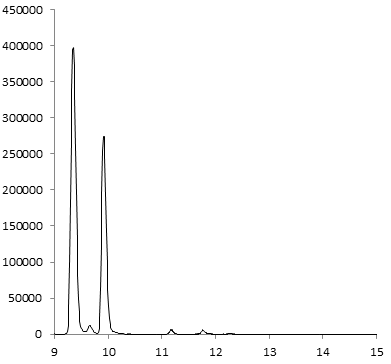


1

2

3

4

5

6

Intensity, CPS

Time (min)

Fig S1 LC–MS extracted total ion chromatogram (TIC) of 6 investigated compounds in FA decoction sample (FA concentration at 250 ug/mL) in the MRM scan mode. naringin (1), hesperidin (2), neohesperidin (3), meranzin hydrate (4), naringenin (5) and hesperetin (6). LC was carried out using a Prominence UFLC XR system (SHIMADZU, LC-20AD), equipped with API 3200 mass spectrometer (AB SCIEX). The mobile phase consisted of methanol (A) and 0.1% formic acid in water (B) using a gradient elution of 10–60% A at 0.01–11 min, 100–100% A at 11.1–15 min. The column temperature was set at 40 °C, the auto-sampler temperature was set at 4 °C. The injection volume was 1 μL and the flow rate was 0.30 mL/min. Typical ion source parameters were: Curtain Gas (CUR)=20, Collision Gas(CAD)=5, IonSpray Voltage(IS)= -4500, Temperature(TEM) = 500 °C, Ion Source Gas 1(GS1)=50, Ion Source Gas 2(GS2)=50.The MS process: Negative at 0-10.305 min, Positive at 10.305-11.305, Negative at 11.305-15 min.

Table S5 The detected MS parameters for the six components.

| Components | RT  (min) | Precursor ion | Product ion | Collision Energy  (CE) | Entrance Potential  (EP) | Collision Cell Exit  Potential (CXP) | Declustering  Potential (DP) |
| --- | --- | --- | --- | --- | --- | --- | --- |
| naringin | 9.31 | 579.0 | 270.9 | -45 | -7 | -1 | -91 |
|  |  | 579.0 | 150.9 | -56 | -7 | -0.5 | -91 |
| hesperidin | 9.62 | 609.0 | 301.0 | -34 | -5 | -1 | -77 |
|  |  | 609.0 | 163.9 | -70 | -4 | -1 | -77 |
| neohesperidin | 9.86 | 609.0 | 301.0 | -47 | -10 | -2 | -80 |
|  |  | 609.0 | 163.9 | -76 | -10 | -2 | -80 |
| meranzin hydrate | 11.20 | 279.0 | 189.1 | 23 | 10 | 2 | 25 |
|  |  | 279.0 | 131.0 | 43 | 9 | 2 | 25 |
| naringenin | 11.74 | 270.9 | 150.9 | -23 | -10 | -2 | -45 |
|  |  | 270.9 | 118.9 | -35 | -10 | -3 | -10 |
| hesperetin | 12.24 | 300.9 | 163.9 | -35 | -10 | -1 | -60 |
|  |  | 300.9 | 150.9 | -34 | -10 | -3 | -60 |
